# Epidemiological analysis of an outbreak of an adenovirus type 7 infection in a boot camp in China

**DOI:** 10.1371/journal.pone.0232948

**Published:** 2020-06-01

**Authors:** Zuiyuan Guo, Libo Tong, Shuang Xu, Bing Li, Zhuo Wang, Yuandong Liu

**Affiliations:** Department of Disease Control, Center for Disease Control and Prevention in Northern Theater Command, Shenyang, Liaoning, China; Defense Threat Reduction Agency, UNITED STATES

## Abstract

**Background:**

An outbreak of respiratory disease associated with adenovirus type 7 occurred in a boot camp in China and was characterized by many cases, severe symptoms, and intrapulmonary infection in many patients.

**Methods:**

We implemented a series of comprehensive preventive and control measures. We analyzed the incubation period and generation time by using the maximum likelihood method, assessed the symptom period and hospitalization duration using the Kaplan-Meier method, and estimated the basic reproductive number and dormitory transmission rate by using established methods.

**Results:**

The epidemic lasted for 30 days, and 375 individuals were affected. Overall, 109 patients were hospitalized, and 266 individuals were isolated and treated. The median incubation period was 5.2 days (95% confidence interval [CI]: 5.0 to 5.4 days). The median generation time was 7.3 days (95% CI: 7.1 to 7.6 days). The median symptom period was 6 days (95% CI: 6 to 7 days). The median hospitalization duration was 9 days (95% CI: 9 to 11 days). The basic reproductive number was 5.1 (95% CI: 4.6 to 5.6), and the dormitory transmission rate was 0.15 (95% CI: 0.12 to 0.18).

**Conclusion:**

Patients in the early stage of the epidemic were treated as having a regular cold and were not isolated; therefore, the virus continued to be transmitted to other susceptible individuals.

## Introduction

Adenoviruses are highly contagious among susceptible human populations and can cause a wide range of clinical symptoms [1–3]. Symptomatic patients are the main source of infection, and in cases of individuals with weak immunity, the virus can be continuously discharged from the body of the infected individual in the absence of clinical symptoms [1, 2]. Infection can be acquired through close contact (such as touching and handshaking) with infected individuals and through the inhalation of aerosols from coughing and sneezing by infected individuals. Humans are generally susceptible to adenoviruses, and people with weak immunity are at a high risk of developing severe symptoms [1, 2]. Currently, only US military personnel receive adenovirus type 4 and type 7 vaccinations; other populations are not immunized with these vaccines [4].

Many countries in the world have experienced adenovirus epidemics [5–8], especially outbreaks of adenovirus type 7, in densely populated settings, such as schools and military installations. These epidemics spread rapidly and affect a substantial portion of the population. Respiratory failure and even death may occur in severe cases [6, 9–14]. In China, there have been numerous reports of adenovirus type 7 outbreaks in the military, especially among new recruits [9–12]. These reports have mainly discussed clinical symptoms and etiology, thereby improving the understanding of the genetic characteristics of pathogens. However, studies describing the characteristics of adenovirus epidemics from the perspective of field and theoretical epidemiology are still lacking. Herein, we clearly describe the measures taken to control an adenovirus type 7 outbreak and quantitatively analyze indicators, such as incubation period, generation time, and basic reproduction number. This study not only accurately describes the epidemiological characteristics of the outbreak but also provides a valuable reference for people dealing with such outbreaks in the future.

## Materials and methods

### Patients

On September 10, 2018, new recruits at a boot camp in Jilin Province, Northeast China, began closed military training. All the trainees and trainers were men. The ages of the trainees ranged mainly between 18 and 22 years, whereas the trainers were older, mostly between 20 and 26 years of age. All of the trainees remained within a closed camp for training, eating, resting, and shopping, and there were no other soldiers, except for the trainees, in the camp. On October 27, individuals presented with symptoms, including high fever, fatigue, systemic pain, and sore throat, and some patients presented with diarrhea. The unit health center sent trainees with a persistent high fever and severe symptoms to a large local hospital for treatment. On November 10, the Chinese Center for Disease Control and Prevention in the Northern Theater Command (Northern Theater CDC) systematically carried out epidemiological investigations and preventive and control measures. These investigations showed that this unit had recruited 835 trainees. In addition, the unit had almost 135 trainers, who were primarily responsible for providing training and performing daily management. All trainees were divided into three districts, each containing 24–27 classes, and each district lived separately in dormitory buildings. Each class, which consisted of 10 trainees, were assigned to a separate dormitory. All trainees dined together in one dining hall.

### Laboratory testing

Investigators randomly collected throat swabs from 40 patients and then used an adenovirus nucleic acid detection kit (JC10117, Bioperfectus Technologies, Jiangsu, China) and influenza type A and type B kits (JC10103 and JC10104, Bioperfectus Technologies) for a real-time fluorescence quantitative polymerase chain reaction assay (qRT-PCR). After adenovirus nucleic acid was detected and influenza virus infection was ruled out, we then used typing detection kits to concurrently assess for the presence of adenovirus types 3, 4, 7, 14, and 55. (Typing primer sets were ordered from Sangon Biotech Co., Ltd., Shanghai, China, according to the primer sets sequences of the above adenovirus types provided by our research group. The primer sets sequences are listed in S5 Table. The one step qRT-PCR kit we used in these detection experiments was the one step qRT-PCR kit (probe) HS0624, UPTECH^TM^ life science, Beijing Hooseen biotechnology Co., Ltd., Beijing, China). Eventually, positive results were obtained for adenovirus type 7 for all of the samples, whereas adenovirus types 3, 4, 14, and 55 were not detected.

### Control measures

On the day the Northern Theater CDC entered the unit to conduct the investigation, an epidemic prevention and control command group composed of the Northern Theater CDC, clinical treatment experts, and unit leaders was established. Under the unified deployment of the command group, medical treatment, health, and epidemic prevention and logistical support teams collaborated closely. Two communication and coordination meetings were held daily to assess the current situation and to deploy the next step of work so that the prevention and control of the epidemic always maintained a flexible and highly efficient information pathway.

Investigators used a four-story vacant dormitory building in the camp as an isolated treatment area to treat patients with a fever below 39°C and relatively mild symptoms. Patients whose body temperature exceeded 39°C or who had severe complications were immediately transferred to the hospital for treatment. A fever clinic was established on the first floor of the isolated treatment area, and all new fever patients were sent to this clinic for initial diagnosis. Patients’ body temperature was measured four times a day. Meals were delivered to patients by special personnel, disposable tableware was used, and the drinking water provided was purchased mineral water. Fresh fruits and milk were provided daily. Psychologists provided psychological counseling for patients. The air was disinfected once daily with chorine-containing solutions. Domestic garbage was collected and burned by special personnel. Because patients might remain infectious after recovery, another four-story vacant dormitory building in the camp was used as an isolation area for recovered patients. Patients from the isolated treatment area and those who were hospitalized were sent to this area after recovery and continued to be isolated and monitored for 7 days.

All exposed individuals who did not develop disease symptoms participated in normal training, and their body temperature was monitored four times a day. Doctors made several rounds of visits to ensure that individuals with a high fever were promptly identified and sent to the fever clinic. The three districts had different dining times, and disinfection of the dining and kitchen utensils was strengthened. The supermarket in the camp was closed to prevent cross-infection. Preventive disinfection of the unit health center, dining hall, dormitories, restrooms, and vehicles specifically allocated for patient transportation was performed daily. Adenovirus prevention and treatment leaflets were produced and issued to all trainees.

### Data collection

Investigators from the Northern Theater CDC conducted a case-by-case investigation. Natural information, which was entered into an Excel spreadsheet, was collected. After the epidemic, the investigators revisited the unit and the hospital to supplement the information already collected and verify the recovery time, discharge time, and chest computed tomography (CT) scan results to ensure the entirety and accuracy of the information; therefore, this investigation had no censored data. The detailed information on the methods and raw data used in the statistical analyses is provided in the supplemental material.

### Incubation period

The incubation period refers to the duration between infection and the appearance of symptoms. Previous studies have shown that the incubation period of most respiratory infections follows a lognormal distribution [15–17]. First, we searched for infector-infectee pairs and investigated the intervals between the infection and the onset. Then, 1000 samples were randomly selected from the two intervals by using the bootstrap method. For each sample, we calculated two parameters for the probability density function according to the maximum likelihood method. A point estimate was acquired by calculating the median of the two parameters.

### Generation time

The generation time refers to the duration between successive onsets of symptoms in a pair with a transmission relationship. Here, following a previous study, we used the Weibull distribution as the probability distribution of the generation time [15]. We determined the disease onset intervals of the infector and the infectee by using the same transmission chain used for calculating the incubation period. Similarly, we randomly sampled from the two intervals by using the bootstrap method and calculated two parameters for the probability density function according to the maximum likelihood method.

### Symptom and hospitalization duration

Symptom and hospitalization duration refer to the durations of the clinical symptoms and hospitalization of patients, respectively. We collected the symptom durations of 107 patients with fever and the hospitalization durations of 109 patients who were hospitalized and performed nonparametric estimations by using the Kaplan-Meier method [18]. In the end, we performed parametric estimations to fit the survival data with the log-logistic function.

### Transmission characteristics

We estimated the basic reproductive number, which refers to the average number of susceptible individuals who were infected by one infector during the entire infection period during the initial stage of the epidemic. This indicator was based on the assumption that the growth rate of the patient number increased exponentially at the beginning of the epidemic [19, 20].

In addition to training outdoors, the trainees spent most of their time in the dormitory resting and studying. To determine the virus transmission efficiency in the dormitory, we estimated the secondary attack rate in the dormitory; this rate was calculated by dividing the number of next-generation patients in a dormitory with at least one patient by the total number of exposed individuals in the same dormitory. Additionally, we also estimated the dormitory transmission rate by using the Reed-Frost method, which refers to the probability of a susceptible individual in the dormitory being infected by exposure to a patient.

### Ethics statements

The Northern Theater CDC is responsible for investigating and controlling public health emergencies. The investigation of the adenovirus type 7 epidemic was a public health concern and, therefore, did not require review by the institutional review board or written informed consent. Respondents’ responses during the investigation were voluntary. Information on patients includes age, class, date of onset, date of treatment, and clinical symptoms. All data were fully anonymized before access.

## Results

### Cause of the epidemic

The unit was under closed management from September 10, 2018, when the training began, until the beginning of the epidemic on October 27. None of the trainees and trainers went outside the camp, nor did they have contact with people outside. We speculate that the infection source of the epidemic was an individual with an asymptomatic infection. Due to a month of high-intensity training and poor adaptation to the boot camp’s cold and dry climate, that individual’s immunity became compromised, and he started shedding infectious virus. During the early stage of the epidemic, the unit health center failed to make a timely diagnosis and did not take effective isolation measures.

### Epidemiological distribution and clinical characteristics

**Fig 1:**
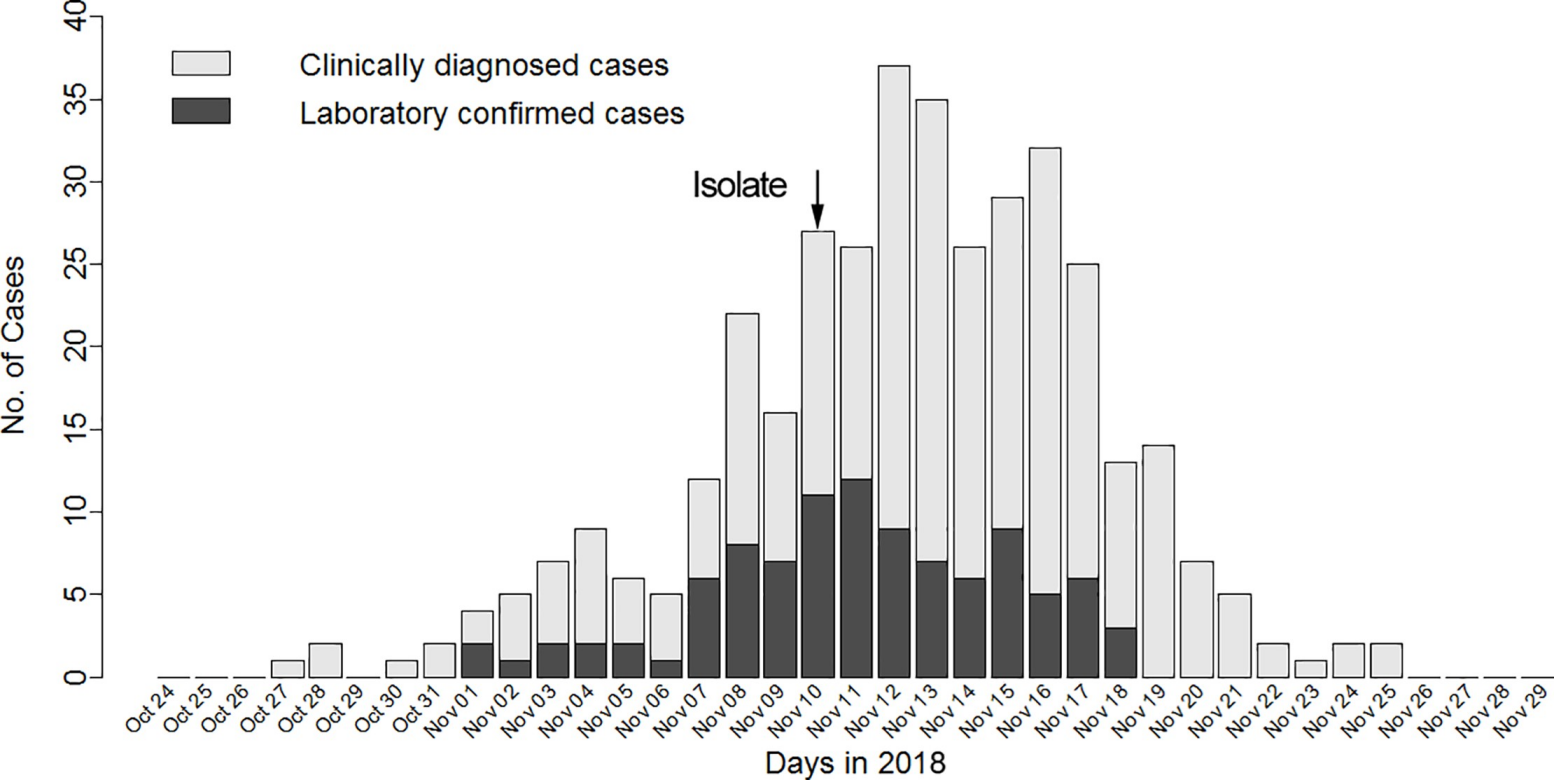
Temporal distribution of new laboratory-confirmed cases and clinically diagnosed cases by day. Legend: Patients with positive viral nucleic acid tests were defined as confirmed cases, and patients who were not tested or whose test results were negative but who exhibited clinical symptoms consistent with adenovirus infection were defined as clinically diagnosed cases.

The first case appeared on October 27 (Fig 1), and then the number of new cases increased gradually, peaking (with 37 new patients) on November 12. From November 17, the number of new cases decreased substantially, and no new patients were seen after November 26.

**Fig 2:**
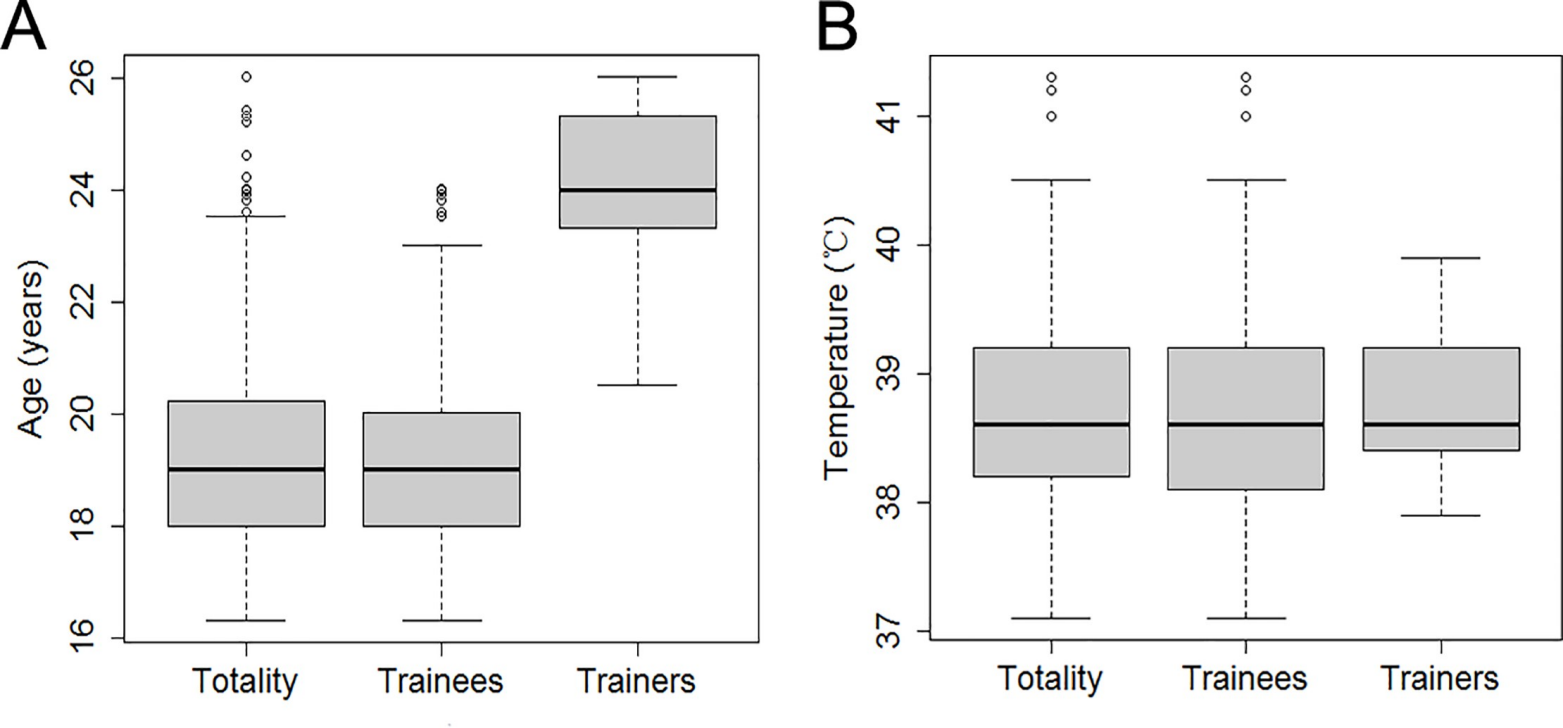
Age distribution and body temperature distribution for the patients. Legend: A. Age distribution of the trainees, the trainers, and the total population. B. Body temperature distribution of the trainees, the trainers, and the total population.

A total of 375 patients were involved in this outbreak. Among them, 99 patients were laboratory confirmed, and 276 patients were clinically diagnosed. A total of 266 patients were treated in the isolated treatment area, and 109 patients were hospitalized. Chest CT examinations showed that 32 patients had an intrapulmonary infection or effusion. Overall, the involved patients included 17 trainers and 358 trainees; the numbers of involved patients consisting of trainers and trainees corresponded to attack rates of 12.6% and 42.9%, respectively. Fisher’s exact test showed that the attack rates for these two groups were significantly different (*p*<0.0001). The attack rates for the first, second, and third district teams were 38.2%, 32.6%, and 44.9%, respectively. A chi-square test showed that the attack rates among the three groups were significantly different (χ^2^ = 10.05, *p* = 0.01). Trainer and trainee ages are shown in Fig 2A.

**Fig 3:**
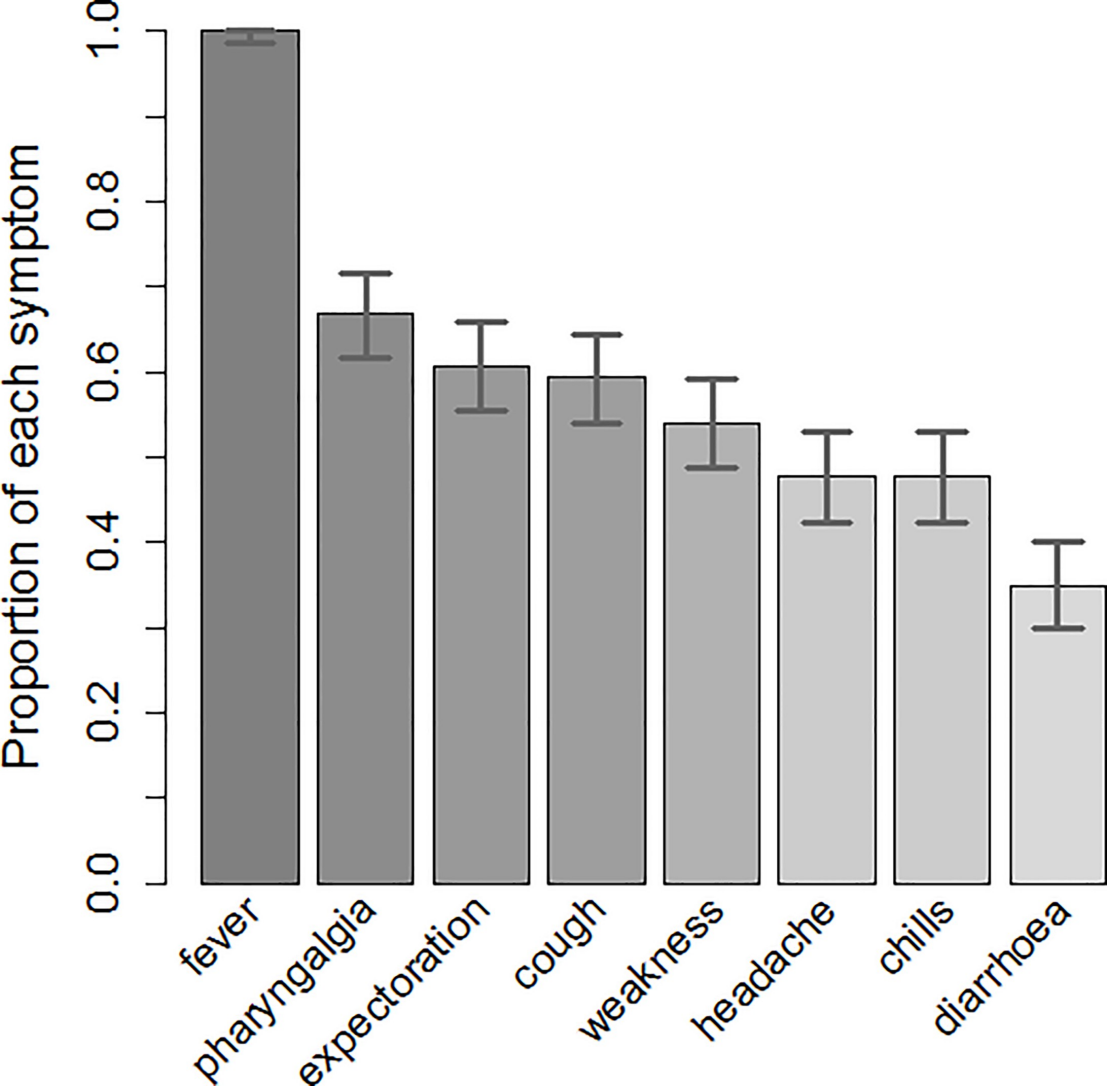
Clinical symptoms. Legend: The percentages for fever, pharyngalgia, expectoration, cough, weakness, headache, chills, and diarrhea were 100%, 67% (95% CI: 62% to 72%), 61% (95% CI: 55% to 66%), 59% (95% CI: 54% to 64%), 54% (95% CI: 49% to 59%), 48% (95% CI: 42% to 53%), 48% (95% CI: 42% to 53%), and 34% (95% CI: 30% to 40%), respectively.

We investigated the clinical symptoms of all patients, and the results are shown in Fig 3. All patients had a fever, and 66.9% had a sore throat. The percentages of patients who experienced coughing with phlegm and without (60.7% and 59.3%, respectively) were similar. The percentage of patients with fatigue was 54.0%. The percentages of patients with headache and chills were the same (47.6%). Furthermore, 34.8% of the patients had diarrhea. The body temperatures of the trainers and trainees are shown in Fig 2B.

### Epidemiological characteristics and transmissibility of adenovirus type 7 infection

**Fig 4:**
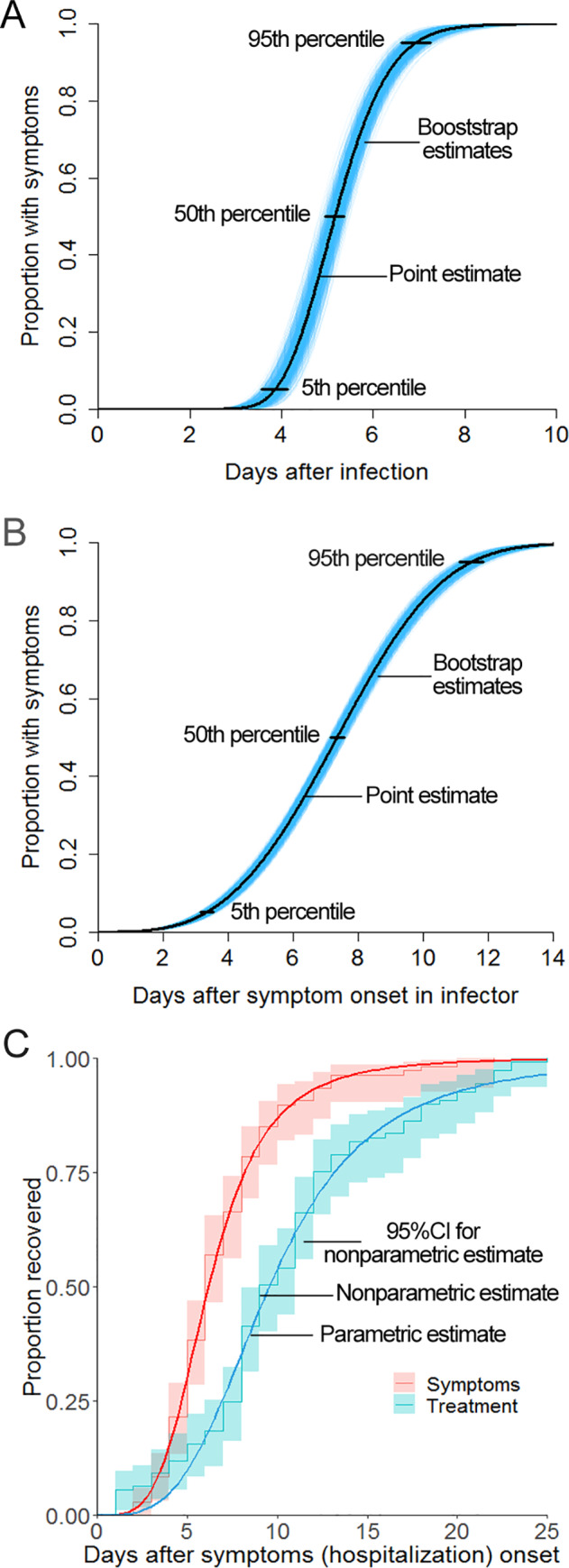
Cumulative probability distributions of the incubation period, generation time, symptom duration, and hospitalization duration. Legend: A. Cumulative probability distribution function of the incubation period. The blue area indicates 1000 cumulative probability distribution function curves with lognormal distribution drawn by the bootstrap method; the point and interval estimations were obtained from the lognormal distribution. B. Cumulative probability distribution function of the generation time. The blue area represents the interval estimate of the Weibull distribution. C. Survival curves of symptom duration and hospitalization duration.

The median incubation period of the adenovirus was 5.2 days (95% CI: 5.0 to 5.4 days) (Fig 4), and 95% of the patients developed symptoms within 6.9 days (95% CI: 6.6 to 7.2 days). The generation time was slightly longer, with a median of 7.3 days (95% CI: 7.1 to 7.6 days), and 95% of the patients developed symptoms within 11.5 days (95% CI: 11.1 to 11.8 days). When a nonparametric method was used for estimation, the median symptom duration was 6 days (95% CI: 6 to 7 days), and 75% of the patients recovered within 8 days (95% CI: 8 to 9 days). The hospitalization duration was 9 days (95% CI: 9 to 11 days), and 75% of the patients were discharged within 12 days (95% CI: 12 to 17 days). When a parametric method was used for estimation, the median symptom duration was 6.2 days, with 75% of patients recovering within 8.2 days, and the median hospitalization duration was 9.6 days, with 75% of patients being discharged within 13.2 days.

A calculation of the attack rate of the entire outbreak estimated that a patient transmitted the disease to an average of 5.1 (95% CI: 4.6 to 5.6) susceptible individuals (basic reproductive number) during the contagious period. The probability of a susceptible individual being infected by contacting a patient (dormitory transmission rate) was 0.15 (95% CI: 0.12 to 0.18). In a dormitory with at least one adenovirus type 7 patient, the secondary attack rate was 0.27 (95% CI: 0.22 to 0.32).

## Discussion

In this epidemic outbreak, the attack rate of the trainees was significantly higher than that of the trainers; this finding is similar to the findings of Lessler and colleagues, who documented that 35% of students and 10% of employees had an influenza-like illness in middle school during a 2009 outbreak of H1N1 influenza [15]; however, the underlying cause for this difference was unclear. We speculate that one reason might be that the trainers had shorter contact with the trainees than the trainees had among themselves; another reason might be that the overall immunity of the trainers was higher than that of the trainees, since trainees felt more stress due to their inadaptation [21]. The attack rates in the three districts were significantly different, and this difference suggests that the severities of the epidemic in the three districts were different. Additionally, cross-infection among trainees of different districts may have occurred, such as during eating, shopping, and showering in public bathrooms.

The median incubation period of this adenovirus infection was 5.2 days; this period is similar to the literature-reported value of 5.6 days [2]; for 95% of patients, the median incubation period was fewer than 7 days. Beginning on November 10, we isolated all new patients and then prevented them from contacting susceptible people. Therefore, most susceptible individuals who were infected before November 10 experienced disease onset during the following 7 days. From November 17, the number of new patients each day decreased rapidly, thus indicating that strict isolation was beneficial for rapid control of the epidemic. The generation time of an epidemic reflects not only a virus’s epidemiological characteristics but also the frequency of contact among people. The median generation time for this epidemic was 7.3 days, the period of which is longer than the median generation time for a typical influenza A (H1N1) outbreak (2.7 days) [15]. The median symptom duration for patients under treatment was 6 days, the period of which was shorter than the hospitalization duration (9 days). This difference is because the patients stayed in the hospital for several days after recovery to ensure complete viral clearance.

We estimated that the basic reproductive number during this epidemic was 5.1, which is higher than the basic reproductive number (1.2) of the swine flu outbreak in New Jersey, USA, in 1976 [22] and the basic reproductive number (3.3) of the influenza A (H1N1) outbreak in New York City in 2009 [15]. The dormitory transmission rate helped further elucidate the efficiency of virus transmission in the dormitory. The rate for this epidemic was 0.15, which was close to the family transmission rate (0.14) of influenza A (H1N1) in 2009 but lower than that (0.32 [23], 0.42 [24]) of interpandemic influenza A. These differences indicate that the dormitory transmission rate for this epidemic was relatively low. A possible reason for the relatively low dormitory transmission rate is that the patients were isolated promptly and, thus, did not contact susceptible individuals; another possible reason is that the concentration of the virus inside the dormitory was low after taking certain measures, such as window ventilation and air disinfection.

By using comprehensive prevention and control measures, the spread of the epidemic was effectively controlled, and all patients recovered. However, there were still some deficiencies in the response to the epidemic. First, at the beginning of the epidemic, the increased patient number did not draw attention from the unit health center; thus, the center did not carry out epidemiological investigations or take effective isolation measures, thereby leading to delays in infection control and to the rapid spread of the infection. Second, some lower-level health workers insufficiently understood epidemiological characteristics and the danger of the adenovirus and other respiratory infectious diseases and were unaware of the importance of early control. Third, no adenovirus-specific vaccines were available. One of the reasons for the high incidence of adenovirus in the Chinese military in recent years is that China has not yet included adenovirus vaccines in planned immunizations.

## Supporting information

S1 Table Time intervals of infection and symptom onset in the patients. Legend: The time intervals of infection for patients Nos. 1–70 were confirmed, and the time intervals of infection for patients Nos. 71–101 were unconfirmed (multiple possibilities).
(DOCX)

S2 Table The time intervals of sequential disease onset for infectors and infectees. Legend: The time intervals of disease onset for infectors Nos. 1–70 were confirmed, and the time intervals of disease onset for infectors Nos. 71–101 were unconfirmed (multiple possibilities).
(DOCX)

S3 Table Symptom and treatment duration (in days) for the patients. (DOCX)

S4 Table Dormitory transmission chain and the number of dormitories in the transmission chain. Legend: *v* represents the number of dormitories, and *I*_*i*_ represents the number of patients at the *i*^*th*^ generation of the transmission chain.
(DOCX)

S5 Table Primers and probes sequences of different types of human adenovirus. (DOCX)

S1 File (DOCX)
