# Supplementary material for: Epidemiological analysis of an outbreak of an adenovirus type 7 infection in a boot camp in China
Source: PLoS One. 2020 Jun 1;15(6):e0232948. doi: 10.1371/journal.pone.0232948 (PMC7263602; doi:10.1371/journal.pone.0232948)
Supplement: S2 Table — Legend: The time intervals of disease onset for infectors Nos. 1–70 were confirmed, and the time intervals of disease onset for infectors Nos. 71–101 were unconfirmed (multiple possibilities). (DOCX) [file pone.0232948.s002.docx]

**S2 Table. Time intervals of sequential disease onset for infectors and infectees.**

| No. | $U_{L}$ | $U_{H}$ | $T_{L}$ | $T_{H}$ | No. | $U_{L}$ | $U_{H}$ | $T_{L}$ | $T_{H}$ |
| --- | --- | --- | --- | --- | --- | --- | --- | --- | --- |
| 1 | Nov.2 | Nov.3 | Nov.10 | Nov.11 | 72 | Nov.9 | Nov.10 | Nov.15 | Nov.16 |
| 2 | Oct.30 | Oct.31 | Nov.4 | Nov.5 |  | Nov.2 | Nov.3 | Nov.15 | Nov.16 |
| 3 | Nov.5 | Nov.6 | Nov.10 | Nov.11 | 73 | Nov.6 | Nov.7 | Nov.10 | Nov.11 |
| 4 | Nov.6 | Nov.7 | Nov.11 | Nov.12 |  | Nov.3 | Nov.4 | Nov.10 | Nov.11 |
| 5 | Nov.5 | Nov.6 | Nov.12 | Nov.13 |  | Nov.2 | Nov.3 | Nov.10 | Nov.11 |
| 6 | Nov.1 | Nov.2 | Nov.9 | Nov.10 | 74 | Nov.2 | Nov.3 | Nov.10 | Nov.11 |
| 7 | Nov.18 | Nov.19 | Nov.29 | Nov.30 |  | Nov.1 | Nov.2 | Nov.10 | Nov.11 |
| 8 | Nov.2 | Nov.3 | Nov.12 | Nov.13 |  | Oct.30 | Oct.31 | Nov.10 | Nov.11 |
| 9 | Nov.1 | Nov.2 | Nov.14 | Nov.15 | 75 | Nov.7 | Nov.8 | Nov.12 | Nov.13 |
| 10 | Nov.5 | Nov.6 | Nov.13 | Nov.14 |  | Nov.6 | Nov.7 | Nov.12 | Nov.13 |
| 11 | Nov.6 | Nov.7 | Nov.15 | Nov.16 |  | Nov.4 | Nov.5 | Nov.12 | Nov.13 |
| 12 | Nov.5 | Nov.6 | Nov.15 | Nov.16 | 76 | Nov.6 | Nov.7 | Nov.14 | Nov.15 |
| 13 | Nov.9 | Nov.10 | Nov.13 | Nov.14 |  | Nov.5 | Nov.6 | Nov.14 | Nov.15 |
| 14 | Nov.8 | Nov.9 | Nov.15 | Nov.16 | 77 | Nov.6 | Nov.7 | Nov.15 | Nov.16 |
| 15 | Nov.2 | Nov.3 | Nov.7 | Nov.8 |  | Nov.5 | Nov.6 | Nov.15 | Nov.16 |
| 16 | Nov.8 | Nov.9 | Nov.13 | Nov.14 | 78 | Nov.8 | Nov.9 | Nov.12 | Nov.13 |
| 17 | Nov.8 | Nov.9 | Nov.13 | Nov.14 |  | Nov.6 | Nov.7 | Nov.12 | Nov.13 |
| 18 | Nov.7 | Nov.8 | Nov.15 | Nov.16 | 79 | Nov.8 | Nov.9 | Nov.13 | Nov.14 |
| 19 | Nov.9 | Nov.10 | Nov.16 | Nov.17 |  | Nov.7 | Nov.8 | Nov.13 | Nov.14 |
| 20 | Nov.9 | Nov.10 | Nov.16 | Nov.17 |  | Nov.5 | Nov.6 | Nov.13 | Nov.14 |
| 21 | Nov.6 | Nov.7 | Nov.11 | Nov.12 | 80 | Nov.8 | Nov.9 | Nov.14 | Nov.15 |
| 22 | Nov.7 | Nov.8 | Nov.14 | Nov.15 |  | Nov.7 | Nov.8 | Nov.14 | Nov.15 |
| 23 | Nov.4 | Nov.5 | Nov.9 | Nov.10 | 81 | Nov.1 | Nov.2 | Nov.10 | Nov.11 |
| 24 | Nov.2 | Nov.3 | Nov.13 | Nov.14 |  | Nov.5 | Nov.6 | Nov.10 | Nov.11 |
| 25 | Nov.2 | Nov.3 | Nov.14 | Nov.15 | 82 | Nov.5 | Nov.6 | Nov.12 | Nov.13 |
| 26 | Nov.6 | Nov.7 | Nov.11 | Nov.12 |  | Nov.1 | Nov.2 | Nov.12 | Nov.13 |
| 27 | Oct.29 | Oct.30 | Nov.10 | Nov.11 | 83 | Nov.8 | Nov.9 | Nov.13 | Nov.14 |
| 28 | Nov.7 | Nov.8 | Nov.12 | Nov.13 |  | Nov.5 | Nov.6 | Nov.13 | Nov.14 |
| 29 | Nov.6 | Nov.7 | Nov.13 | Nov.14 |  | Nov.1 | Nov.2 | Nov.13 | Nov.14 |
| 30 | Nov.7 | Nov.8 | Nov.16 | Nov.17 | 84 | Nov.7 | Nov.8 | Nov.14 | Nov.15 |
| 31 | Nov.7 | Nov.8 | Nov.16 | Nov.17 |  | Nov.6 | Nov.7 | Nov.14 | Nov.15 |
| 32 | Nov.10 | Nov.11 | Nov.15 | Nov.16 |  | Nov.5 | Nov.6 | Nov.14 | Nov.15 |
| 33 | Nov.10 | Nov.11 | Nov.16 | Nov.17 |  | Nov.4 | Nov.5 | Nov.14 | Nov.15 |
| 34 | Nov.1 | Nov.2 | Nov.11 | Nov.12 | 85 | Nov.6 | Nov.7 | Nov.10 | Nov.11 |
| 35 | Nov.1 | Nov.2 | Nov.12 | Nov.13 |  | Nov.4 | Nov.5 | Nov.10 | Nov.11 |
| 36 | Nov.1 | Nov.2 | Nov.12 | Nov.13 | 86 | Nov.10 | Nov.11 | Nov.15 | Nov.16 |
| 37 | Nov.9 | Nov.10 | Nov.14 | Nov.15 |  | Nov.7 | Nov.8 | Nov.15 | Nov.16 |
| 38 | Nov.9 | Nov.10 | Nov.14 | Nov.15 | 87 | Nov.10 | Nov.11 | Nov.15 | Nov.16 |
| 39 | Nov.9 | Nov.10 | Nov.15 | Nov.16 |  | Nov.9 | Nov.10 | Nov.15 | Nov.16 |
| 40 | Nov.2 | Nov.3 | Nov.10 | Nov.11 | 88 | Nov.11 | Nov.12 | Nov.15 | Nov.16 |
| 41 | Nov.2 | Nov.3 | Nov.11 | Nov.12 |  | Nov.10 | Nov.11 | Nov.15 | Nov.16 |
| 42 | Nov.2 | Nov.3 | Nov.12 | Nov.13 | 89 | Nov.9 | Nov.10 | Nov.14 | Nov.15 |
| 43 | Nov.2 | Nov.3 | Nov.12 | Nov.13 |  | Nov.8 | Nov.9 | Nov.14 | Nov.15 |
| 44 | Nov.1 | Nov.2 | Nov.7 | Nov.8 |  | Nov.2 | Nov.3 | Nov.14 | Nov.15 |
| 45 | Nov.1 | Nov.2 | Nov.10 | Nov.11 | 90 | Nov.6 | Nov.7 | Nov.12 | Nov.13 |
| 46 | Nov.9 | Nov.10 | Nov.14 | Nov.15 |  | Nov.2 | Nov.3 | Nov.12 | Nov.13 |
| 47 | Nov.9 | Nov.10 | Nov.13 | Nov.14 | 91 | Nov.8 | Nov.9 | Nov.14 | Nov.15 |
| 48 | Nov.5 | Nov.6 | Nov.9 | Nov.10 |  | Nov.5 | Nov.6 | Nov.14 | Nov.15 |
| 49 | Nov.6 | Nov.7 | Nov.13 | Nov.14 | 92 | Nov.11 | Nov.12 | Nov.15 | Nov.16 |
| 50 | Nov.3 | Nov.4 | Nov.12 | Nov.13 |  | Nov.9 | Nov.10 | Nov.15 | Nov.16 |
| 51 | Nov.4 | Nov.5 | Nov.13 | Nov.14 |  | Nov.6 | Nov.7 | Nov.15 | Nov.16 |
| 52 | Nov.9 | Nov.10 | Nov.15 | Nov.16 | 93 | Nov.10 | Nov.11 | Nov.15 | Nov.16 |
| 53 | Nov.7 | Nov.8 | Nov.15 | Nov.16 |  | Nov.8 | Nov.9 | Nov.15 | Nov.16 |
| 54 | Nov.9 | Nov.10 | Nov.15 | Nov.16 |  | Nov.5 | Nov.6 | Nov.15 | Nov.16 |
| 55 | Nov.8 | Nov.9 | Nov.15 | Nov.16 | 94 | Nov.7 | Nov.8 | Nov.11 | Nov.12 |
| 56 | Nov.7 | Nov.8 | Nov.11 | Nov.12 |  | Nov.5 | Nov.6 | Nov.11 | Nov.12 |
| 57 | Oct.29 | Oct.30 | Nov.1 | Nov.2 | 95 | Nov.7 | Nov.8 | Nov.12 | Nov.13 |
| 58 | Oct.30 | Oct.31 | Nov.11 | Nov.12 |  | Nov.6 | Nov.7 | Nov.12 | Nov.13 |
| 59 | Nov.10 | Nov.11 | Nov.14 | Nov.15 |  | Nov.4 | Nov.5 | Nov.12 | Nov.13 |
| 60 | Nov.9 | Nov.10 | Nov.15 | Nov.16 | 96 | Nov.7 | Nov.8 | Nov.13 | Nov.14 |
| 61 | Oct.29 | Oct.30 | Nov.3 | Nov.4 |  | Nov.6 | Nov.7 | Nov.13 | Nov.14 |
| 62 | Oct.29 | Oct.30 | Nov.4 | Nov.5 |  | Nov.4 | Nov.5 | Nov.13 | Nov.14 |
| 63 | Oct.29 | Oct.30 | Nov.4 | Nov.5 | 97 | Nov.8 | Nov.9 | Nov.15 | Nov.16 |
| 64 | Oct.29 | Oct.30 | Nov.5 | Nov.6 |  | Nov.6 | Nov.7 | Nov.15 | Nov.16 |
| 65 | Oct.29 | Oct.30 | Nov.5 | Nov.6 | 98 | Nov.11 | Nov.12 | Nov.16 | Nov.17 |
| 66 | Oct.30 | Oct.31 | Nov.6 | Nov.7 |  | Nov.9 | Nov.10 | Nov.16 | Nov.17 |
| 67 | Nov.4 | Nov.5 | Nov.16 | Nov.17 | 99 | Nov.9 | Nov.10 | Nov.15 | Nov.16 |
| 68 | Nov.7 | Nov.8 | Nov.13 | Nov.14 |  | Nov.6 | Nov.7 | Nov.15 | Nov.16 |
| 69 | Nov.4 | Nov.5 | Nov.8 | Nov.9 | 100 | Nov.10 | Nov.11 | Nov.16 | Nov.17 |
| 70 | Nov.3 | Nov.4 | Nov.9 | Nov.10 |  | Nov.7 | Nov.8 | Nov.16 | Nov.17 |
| 71 | Nov.2 | Nov.3 | Nov.13 | Nov.14 | 101 | Nov.4 | Nov.5 | Nov.14 | Nov.15 |
|  | Nov.8 | Nov.9 | Nov.13 | Nov.14 |  | Nov.3 | Nov.4 | Nov.14 | Nov.15 |

Legend: The time intervals of disease onset for infectors Nos. 1-70 were confirmed, and the time intervals of disease onset for infectors Nos. 71-101 were unconfirmed (multiple possibilities).
