# Supplementary material for: Epidemiological analysis of an outbreak of an adenovirus type 7 infection in a boot camp in China
Source: PLoS One. 2020 Jun 1;15(6):e0232948. doi: 10.1371/journal.pone.0232948 (PMC7263602; doi:10.1371/journal.pone.0232948)
Supplement: S3 Table — (DOCX) [file pone.0232948.s003.docx]

**S3 Table. Symptom and treatment duration (in days) for the patients.**

| No. | Symptom period | Treatment period | No. | Symptom period | Treatment period |
| --- | --- | --- | --- | --- | --- |
| 1 | 2 | 1 | 55 | 6 | 9 |
| 2 | 2 | 1 | 56 | 6 | 10 |
| 3 | 2 | 1 | 57 | 6 | 10 |
| 4 | 3 | 1 | 58 | 6 | 10 |
| 5 | 3 | 1 | 59 | 6 | 10 |
| 6 | 3 | 1 | 60 | 6 | 11 |
| 7 | 3 | 2 | 61 | 6 | 11 |
| 8 | 3 | 3 | 62 | 7 | 11 |
| 9 | 3 | 3 | 63 | 7 | 11 |
| 10 | 4 | 3 | 64 | 7 | 11 |
| 11 | 4 | 4 | 65 | 7 | 11 |
| 12 | 4 | 4 | 66 | 7 | 11 |
| 13 | 4 | 4 | 67 | 7 | 11 |
| 14 | 4 | 5 | 68 | 7 | 11 |
| 15 | 4 | 5 | 69 | 7 | 11 |
| 16 | 4 | 5 | 70 | 7 | 11 |
| 17 | 4 | 5 | 71 | 7 | 11 |
| 18 | 4 | 6 | 72 | 8 | 11 |
| 19 | 4 | 6 | 73 | 8 | 12 |
| 20 | 4 | 6 | 74 | 8 | 12 |
| 21 | 4 | 7 | 75 | 8 | 12 |
| 22 | 4 | 7 | 76 | 8 | 12 |
| 23 | 4 | 7 | 77 | 8 | 12 |
| 24 | 5 | 7 | 78 | 8 | 12 |
| 25 | 5 | 7 | 79 | 8 | 12 |
| 26 | 5 | 7 | 80 | 8 | 12 |
| 27 | 5 | 7 | 81 | 8 | 12 |
| 28 | 5 | 8 | 82 | 8 | 12 |
| 29 | 5 | 8 | 83 | 8 | 13 |
| 30 | 5 | 8 | 84 | 8 | 13 |
| 31 | 5 | 8 | 85 | 9 | 13 |
| 32 | 5 | 8 | 86 | 9 | 13 |
| 33 | 5 | 8 | 87 | 9 | 14 |
| 34 | 5 | 8 | 88 | 9 | 14 |
| 35 | 5 | 8 | 89 | 9 | 14 |
| 36 | 5 | 8 | 90 | 9 | 15 |
| 37 | 5 | 8 | 91 | 9 | 16 |
| 38 | 5 | 8 | 92 | 10 | 17 |
| 39 | 5 | 8 | 93 | 10 | 17 |
| 40 | 5 | 8 | 94 | 10 | 17 |
| 41 | 5 | 8 | 95 | 10 | 18 |
| 42 | 6 | 8 | 96 | 10 | 18 |
| 43 | 6 | 8 | 97 | 11 | 18 |
| 44 | 6 | 8 | 98 | 12 | 18 |
| 45 | 6 | 8 | 99 | 12 | 19 |
| 46 | 6 | 9 | 100 | 12 | 20 |
| 47 | 6 | 9 | 101 | 13 | 20 |
| 48 | 6 | 9 | 102 | 13 | 21 |
| 49 | 6 | 9 | 103 | 13 | 21 |
| 50 | 6 | 9 | 104 | 17 | 22 |
| 51 | 6 | 9 | 105 | 18 | 22 |
| 52 | 6 | 9 | 106 | 20 | 22 |
| 53 | 6 | 9 | 107 | 22 | 23 |
| 54 | 6 | 9 | 108 |  | 23 |
|  |  |  | 109 |  | 25 |
