# Supplementary material for: Epidemiological analysis of an outbreak of an adenovirus type 7 infection in a boot camp in China
Source: PLoS One. 2020 Jun 1;15(6):e0232948. doi: 10.1371/journal.pone.0232948 (PMC7263602; doi:10.1371/journal.pone.0232948)
Supplement: S4 Table — Legend: v represents the number of dormitories, and Ii represents the number of patients at the ith generation of the transmission chain. (DOCX) [file pone.0232948.s004.docx]

**S4 Table. Dormitory transmission chain and number of dormitories in the transmission chain.**

| $v$ | $I_{0}$ | $I_{1}$ | $I_{2}$ | $I_{3}$ |
| --- | --- | --- | --- | --- |
| 27 | 1 | 0 | 0 | 0 |
| 10 | 1 | 1 | 0 | 0 |
| 6 | 1 | 2 | 0 | 0 |
| 3 | 1 | 3 | 0 | 0 |
| 1 | 1 | 4 | 0 | 0 |
| 6 | 2 | 2 | 0 | 0 |
| 1 | 2 | 6 | 0 | 0 |
| 1 | 3 | 1 | 0 | 0 |
| 1 | 3 | 2 | 0 | 0 |
| 1 | 4 | 1 | 0 | 0 |
| 2 | 1 | 1 | 2 | 0 |
| 1 | 1 | 1 | 3 | 0 |
| 1 | 1 | 1 | 5 | 0 |
| 1 | 1 | 2 | 1 | 0 |
| 1 | 1 | 2 | 2 | 0 |
| 1 | 1 | 1 | 1 | 2 |
| 1 | 1 | 1 | 2 | 2 |

Legend: *v* represents the number of dormitories, and *I_i_* represents the number of patients at the *i^th^* generation of the transmission chain.
