# Supplementary material for: Epidemiological analysis of an outbreak of an adenovirus type 7 infection in a boot camp in China
Source: PLoS One. 2020 Jun 1;15(6):e0232948. doi: 10.1371/journal.pone.0232948 (PMC7263602; doi:10.1371/journal.pone.0232948)
Supplement: S5 Table — (DOCX) [file pone.0232948.s005.docx]

**S5 Table. Primers and probes sequences of different types of human adenovirus.**

| Types of human adenovirus | Typing primer sets of HAdV | Sequences |
| --- | --- | --- |
| Human adenovirus type 3 | qHAdV3-F | GGGAGACAATATTACTAAAGAAGGTTTGC |
|  | qHAdV3-R | CAACTTGAGGCTCTGGCTGATA |
|  | qHAdV3-Probe | FAM-CACTAC"T*"GAAGGAGAAGAAAAGCCCA TTTATGCC |
| Human adenovirus type 4 | qHAdV4-F | AAGCCAACCTGTGGAGKAACT |
|  | qHAdV4-R | TGTTGGCCGGTGTGTATTTG |
|  | qHAdV4-Probe | FAM-CTSTATGCCAATGTTGCCCTCTATTTGCC T-BHQ1 |
| Human adenovirus type 7 | qHAdV7-R | AATTGACATTTTCCGTGTAAAGCA |
|  | qHAdV7-Probe | FAM-AAGCTGCTGACGCTTTTTCGCCTGA-BHQ1 |
| Human adenovirus type 14 | qHAdV14-F | GAAAATCATGGTGTGGAAGATGAA |
|  | qHAdV14-R | CAAGCTTGGTCTCCATTTAACTGA |
|  | qHAdV14-Probe | FAM-ACGGCATCGGTCCGCGAACA-BHQ1 |
| Human adenovirus type 11/55 | qHAdV55-F | CGGAGCAGCCAAATCAGAA |
|  | qHAdV55-R | CATGAGTGTCTGGAGTTTCCAAAT |
|  | qHAdV55-Probe | FAM-TGCGGCATCACAGAAAACAAACTTAAG  TC-BHQ1 |
