# Supplementary material for: Epidemiological analysis of an outbreak of an adenovirus type 7 infection in a boot camp in China
Source: PLoS One. 2020 Jun 1;15(6):e0232948. doi: 10.1371/journal.pone.0232948 (PMC7263602; doi:10.1371/journal.pone.0232948)
Supplement: S1 File — (DOCX) [file pone.0232948.s006.docx]

**Supplemental material for** **“Epidemiological analysis of an outbreak of an adenovirus type 7 infection in a boot camp in China”**

Zuiyuan Guo, Libo Tong, ShuangXu, Yuandong Liu

Herein, we elaborate on the statistical methods provided in the methods section of the main text.

**1. Incubation period**

The incubation period refers to the period from infection to the initial onset of symptoms. According to published literature, the incubation period *t* of respiratory infections mostly fits a lognormal (log-norm) distribution $\log t\sim N(\mu,\sigma)$[1-3]; the probability density function is shown in formula 1.

$f(t)=\frac{1}{\sqrt{2\pi}\sigma t}e^{-\frac{(\log t-\mu)^{2}}{2\sigma^{2}}}$ $t>0$ (1)

where $\mu$ and $\sigma$ represent the mean and standard deviation, respectively. We could only estimate the time ranges of infection and the initial onset of symptoms during the investigation, as the time when the event occurred could not be accurately determined. The range of time *s* of infection was set as $(V_{L},V_{H})$, and the range of time *t* when symptoms appeared was set as $(T_{L},T_{H})$. The probability of infection of and symptoms onset in the *i*^th^ patient can be calculated by formula 2:

$h_{i}(\mu,\sigma;V_{iL},V_{iH},T_{iL},T_{iH})=\int_{T_{iL}}^{T_{iH}} \int_{V_{iL}}^{V_{iH}} g(s)f_{\mu,\sigma}(t-s)dsdt$ $i=1,2,\cdots\cdots,N$ (2)

where $g()$ is the probability of infection and *N* is the total number of patients. We assumed that the infection was uniformly distributed within the time period $s\sim U(V_{L},V_{H})$. During the investigation, we confirmed the transmission chain of the epidemic, including 101 infector-infectee pairs, and determined the time range of infection and symptom onset in the infectee in each pair. We performed a round of random sampling on all pairs and used the maximum likelihood method to establish the maximum likelihood function of the probability of disease onset after infection in *N*=101 patients:

$L(\mu,\sigma)=\prod_{i=1}^{N} h_{i}(\mu,\sigma;V_{iL},V_{iH},T_{iL},T_{iH})$ (3)

We used the logarithm of formula 3 and calculated the partial derivatives of *μ* and *σ* to obtain the estimated values for two parameters during a round of random sampling results to further obtain the cumulative probability curve. We performed 1000 rounds of random sampling and obtained 1000 curves for the cumulative probability distribution function; the medians of the two parameters were $\mu\approx1.64$ and $\sigma\approx0.18$.

Because the transmission relationships of some infector/infectee pairs were uncertain, some patients might have had multiple possible infection times. When we performed random sampling on these patients, we randomly selected only one possible infection time. The ranges of the patients’ infection times and symptom onset times are shown in Table S1.

**2. Generation time**

The generation time refers to the time between successive onsets of symptoms in an infector-infectee pair with a transmission relationship. Based on 101 infector-infectee pairs, we can find the time range in which the infector first experienced symptoms $(GV_{L},GV_{H})$. According to a previous study, the Weibull distribution (formula 4) reflects the actual distribution for the generation time [1]. We, therefore, used the Weibull distribution to estimate the generation time:

$f(t)=\frac{mt^{m-1}}{\eta^{m}}e^{-(\frac{t}{\eta})^{m}}$ $t>0$ (4)

where $m$ and $\eta$ represent the shape and scale parameters, respectively; the estimation method for the parameters was similar to that for the incubation period. The time when the infector first exhibited symptoms was also within a certain interval, and we assumed that this time fit a uniform distribution. We first established a probability equation for the successive appearance of symptoms in an infector-infectee pair and then performed a round of random sampling in all of the samples. The maximum likelihood method was used to establish the maximum likelihood function, and we finally calculated the parameter estimates for the round of random sampling via the optim() function in R. After 1000 rounds of random sampling, we found that the median parameters were $shape\approx3.48$ and $scale\approx8.19$.

Some infectees might correspond to multiple possible infectors; therefore, the generation time of these pairs have multiple possibilities. During the random sampling, we randomly selected only one possible onset time of infection. The intervals for the onset time for the infector-infectee pairs are shown in Table S2.

**3. Symptom and hospitalization duration**

Symptom and hospitalization duration refer to the durations of the clinical symptoms and hospitalization of the patients, respectively. We calculated the symptom durations of 107 patients under treatment and the hospitalization duration of 109 patients who were hospitalized. The ggplot2, ggfortify, survival, and survminer packages were applied in R. The survival curve of the nonparametric Kaplan-Meier method and its confidence interval were plotted via the autoplot() function. Additionally, we used a parametric method to fit the survival curves for symptom and hospitalization duration by using a log-logistic distribution, Weibull distribution and gamma distribution (by using the least-squares method) and found that the log-logistic function fit the survival curves best. Finally, we used stat_function() to draw the loglogistic function curve. The symptom and hospitalization durations of the patients are shown in Table S3.

**4. Basic reproductive number**

The number of new patients increased exponentially during the early stage of the epidemic; this increase is indicated by growth rate $\beta$. Based on this characteristic, we estimated the basic reproductive number. Formula 5 can be used for estimation when the generation time follows a gamma distribution.

$R=(1+\beta\mu\upsilon^{2})^{\frac{1}{\upsilon^{2}}}$ (5)

where $\mu$ is the mean generation time and $\upsilon$ is the coefficient of variation. We fitted a gamma distribution of the generation time data, and the mean value and standard deviation were 7.34 and 2.43, respectively. In comparison, the parameters of the Weibull distribution are 7.36 and 2.47. We estimated $\beta$ by using formula 6:

$L(\beta|C)\propto\exp(-\beta\sum_{i=0}^{t-1} C(i))(1-\exp(-\beta))^{C(t)-C(0)}$ (6)

where $C(i)$ is the cumulative number of cases at time $i$. We estimated that the exponential growth period was from October 27 to November 12, 2018. The basic reproductive number was calculated after $\beta$ was acquired by using the maximum likelihood method. The supported interval of *R* was calculated based on the interval of $\beta$ according to the maximum likelihood method.

**5. Dormitory transmission rate**

Dormitory transmission rate *p* refers to the probability of a susceptible individual in a dormitory being infected by contacting a patient, and the probability of not being infected was 1-*p*. The probability of a susceptible individual being infected by contacting *m* patients was $r_{m}=1-(1-p)^{m}$. The probability of *k* susceptible individuals being infected and getting sick when there were *m* patients and *N* susceptible individuals can be expressed by the following binomial theorem:

$pr(I_{t+1}=k|I_{t}=m,S_{t}=N)=\left(\begin{matrix} N \\ k \end{matrix}\right)r_{m}^{k}(1-r_{m})^{N-k}$ $m\geq1,k\geq0$ (7)

where $I_{t}$ and $S_{t}$ represent the number of patients and susceptible individuals, respectively, at the *t*^th^ generation in the transmission chain. In the dormitory, when the transmission chain continued, $I_{0}\to I_{1}\to\cdots\to I_{h}$, until the *h*^th^ generation, the probability of infection was calculated using the following function:

(8)

The number of dormitories with the same transmission chain (i.e., the same number of generations and the same number of individuals being infected in each generation) is represented by $v$, and the probability is represented by $\theta$. We obtained the maximum likelihood function by multiplying the probability of all dormitories using the maximum likelihood method (formula 9).

$L(\theta_{0},\theta_{1},\cdots,\theta_{n})=\theta_{0}^{v1}\cdot\theta_{1}^{v2}\cdot\theta_{2}^{v3}\cdot\cdots\cdot\theta_{n}^{vn}$ (9)

Finally, we calculated the *p* value by using the optim() function in R. The transmission chain, patient number and dormitory number are shown in Table S4.

**References**

1. Lessler J, Reich NG, Cumming DAT. Outbreak of 2009 Pandemic Influenza A (H1N1) at a New York City School. N Engl J Med 2009;361:2628-36.

2. Lessler J, Reich NG, Brookmeyer R, Perl TM, NelsonKE, Cummings DAT. Incubation periods of acute respiratory viral infections: a systematic review. Lancet Infect Dis 2009;9:291–300.

3. Reich NG, Lessler J, Cummings DAT, Brookmeyer R. Estimating incubation period distributions with coarse data. Stat Med 2009;28:2769-84.

4. Becker N. Analysis of Infectious Disease Data. London: Chapman and Hall,1989.
